# Star Memristive Neural Network: Dynamics Analysis, Circuit Implementation, and Application in a Color Cryptosystem

**DOI:** 10.3390/e25091261

**Published:** 2023-08-25

**Authors:** Sen Fu, Zhengjun Yao, Caixia Qian, Xia Wang

**Affiliations:** 1 College of Materials Science and Technology, Nanjing University of Aeronautics and Astronautics, Nanjing 211100, China; 2 Aircraft Technology Branch of Hunan Aerospace Co., Ltd., Changsha 410000, China; 3 China Aerospace Science and Industry Corporation, Beijing 100048, China

**Keywords:** memristor, Hopfield neural network, multi-scroll attractors, initial boosting behavior, circuit implementation, image encryption

## Abstract

At present, memristive neural networks with various topological structures have been widely studied. However, the memristive neural network with a star structure has not been investigated yet. In order to investigate the dynamic characteristics of neural networks with a star structure, a star memristive neural network (SMNN) model is proposed in this paper. Firstly, an SMNN model is proposed based on a Hopfield neural network and a flux-controlled memristor. Then, its chaotic dynamics are analyzed by using numerical analysis methods including bifurcation diagrams, Lyapunov exponents, phase plots, Poincaré maps, and basins of attraction. The results show that the SMNN can generate complex dynamical behaviors such as chaos, multi-scroll attractors, and initial boosting behavior. The number of multi-scroll attractors can be changed by adjusting the memristor’s control parameters. And the position of the coexisting chaotic attractors can be changed by switching the memristor’s initial values. Meanwhile, the analog circuit of the SMNN is designed and implemented. The theoretical and numerical results are verified through MULTISIM simulation results. Finally, a color image encryption scheme is designed based on the SMNN. Security performance analysis shows that the designed cryptosystem has good security.

## 1. Introduction

Because of its rich chaotic dynamics, Hopfield neural networks (HNNs) have attracted much attention from chaos scholars [1,2,3]. It is well known that the human brain nervous system has abundant chaotic discharge behaviors [4], which are closely related to advanced intelligence. HNNs are considered to be the best example of studying the dynamics of the brain’s nervous system [5,6]. At the same time, many researchers have found that neural networks with chaotic behavior have a wide range of applications in many fields including associative memory, pattern recognition, and combinatorial optimization [7,8]. Therefore, the study of the chaotic behavior of HNNs has important theoretical and practical significance for brain science and artificial intelligence.

Since the HNN was proposed, various HNNs have been proposed and their chaotic behaviors studied. For example, normal chaos [9,10,11] and hyperchaos [12,13,14] have been detected in some small HNNs with several neurons. Especially in recent years, memristive neural networks have received much attention from many scholars [15,16]. For example, Pham et al. [17] proposed a three-neuron memristive neural network and revealed its hidden chaotic behavior. Bao et al. [18] found that a memristive neural network consisting of three neurons can produce coexisting asymmetric attractors. A memristive neural network with complex chaotic attractors was proposed by Yu et al. in [19]. Furthermore, plenty of similar works have been reported [20,21,22]. To sum up, these studies are mainly focused on the chaotic dynamics of neural networks with mixed structures. Very recently, Lin et al. [23] proposed a memristive neural network with a ring structure, and complex hyperchaos was discovered. However, there are fewer concerns about the chaotic dynamics of neural networks with a star structure.

The multi-scroll attractor is a complex chaos phenomenon [24,25,26], which has irregular scroll trajectories. Multi-scroll attractors have higher tunability and complexity than single-scroll attractors. The multi-scroll attractors of the MNN were first revealed in Ref. [27]. Wan et al. [28] found multi-double-scroll attractors in the MHNN. In addition, grid multi-scroll attractors have been obtained from an MHNN with two memristor synapses [29]. Meanwhile, some similar memristive neural networks with multi-scroll attractors have been investigated and successfully applied in the information security field [30,31,32].

Initial boosting behavior is a kind of complex behavior that shows extreme multistability [33,34]. Generally, this phenomenon has infinite coexisting attractors with the same shape and different positions [35]. More importantly, the initial offset boosting behavior can provide sustained and robust boosted chaotic sequences and their oscillating amplitudes can be non-destructively controlled by switching the initial states flexibly. These merits make them more practical for chaos engineering applications [36,37]. In recent years, some scholars have been devoted to the initial boosting behavior in neural networks [38,39]. Especially, the initial offset boosting coexisting chaos was observed in a two-memristor-based Hopfield neural network [40]. Hyperchaotic initial offset boosting behavior was revealed in a memristive coupling neural network [41].

This paper investigates the chaotic dynamics of the star memristive neural network and its application in color image encryption. To the best of our knowledge, this is the first time that the chaotic dynamics of the star neural network has been investigated. The main novelty and contributions of this study are summarized as follows. (1) We propose a star memristive neural network model based on one Hopfield neural network with four neurons and one memristor. (2) The star memristive neural network exhibits rich and complex multi-scroll attractors and initial boosting dynamics. (3) A star memristive neural network circuit is realized and numerical simulation results are further demonstrated. (4) We design a color image encryption cryptosystem by using the multi-scroll sequences to show the practical application of the presented star memristive neural network.

The rest of this paper is organized as follows. A star memristive neural network model is constructed in Section 2. The chaotic dynamics of the star memristive neural network is studied in Section 3. An analog circuit of the star memristive neural network is implemented in Section 4. An SMNN-based color image encryption scheme is designed in Section 5, and its security performances are analyzed. The full text is summarized in Section 6.

## 2. Star Memristive Neural Network

### 2.1. Brief Introduction of the Memristor Model

**Figure 1:**
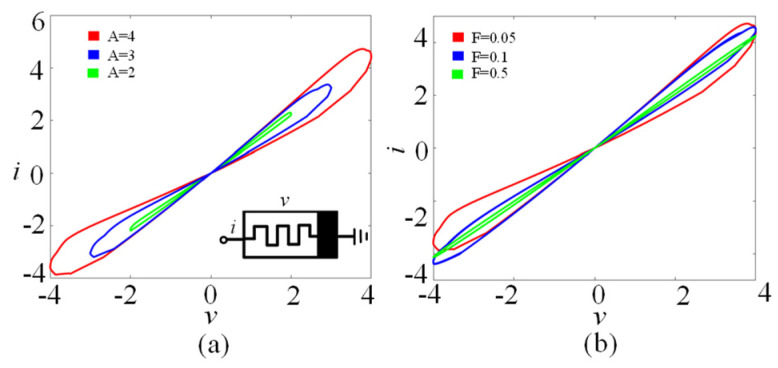
The fingerprints of the memristor driven by *v* = *A*sin(2*πFt*). (**a**) Amplitude-related voltage–current loci for *A* = 2, 3, and 4 with *F* = 0.05 and *x*_0_ = 0. (**b**) Frequency-related voltage–current loci for *F* = 0.05, 0.1, and 0.5 with *A* = 4 and *x*_0_ = 0.

Memristors are usually used to describe electromagnetic induction in the nervous system caused by electromagnetic radiation. Here, a flux-controlled multi-segment nonlinear memristor is introduced [15]. Its mathematical model can be written by

(1) $$\left\{ \begin{array}{c} i=W(\varphi )v\\ W(\varphi )=(a+b\varphi )\\ d\varphi /dt=cv-dh(\varphi ) \end{array}\right.$$

(2) $$h(\varphi )=\{\begin{array}{c} h_{1}(\varphi )=\{\begin{array}{c} \varphi ,N=0\\ \varphi -\sum_{i=1}^{N}\left(\mathrm{sgn}(\varphi +(2i-1))+\mathrm{sgn}(\varphi -(2i-1))\right)\\ N=1,2,3,\ldots \end{array}\\ h_{2}(\varphi )=\{\begin{array}{c} \varphi -\mathrm{sgn}(\varphi ),M=0\\ \varphi -\mathrm{sgn}(\varphi )-\sum_{j=1}^{M}\left(\mathrm{sgn}(\varphi +2j)+\mathrm{sgn}(\varphi -2j)\right)\\ M=1,2,3,\ldots \end{array} \end{array}$$

where *i*, *v*, and *φ* are the current, voltage, and state variables of the memristor, respectively. The function *h*(*φ*) is the state function of the memristor, where *N* and *M* are two control parameters. The memristor model can exhibit typical memristive characteristics, as shown in Figure 1. As can be seen from Figure 1a, the memristor model can generate three tight hysteresis loops under different voltage amplitudes (*A* = 1, 2, 3). Figure 1b shows that with the increase in voltage frequency (*F* = 0.05, 0.1, 0.5), the area of the tight hysteresis loop of the memristor decreases gradually.

### 2.2. Memristive Star Neural Network Modeling

**Figure 2:**
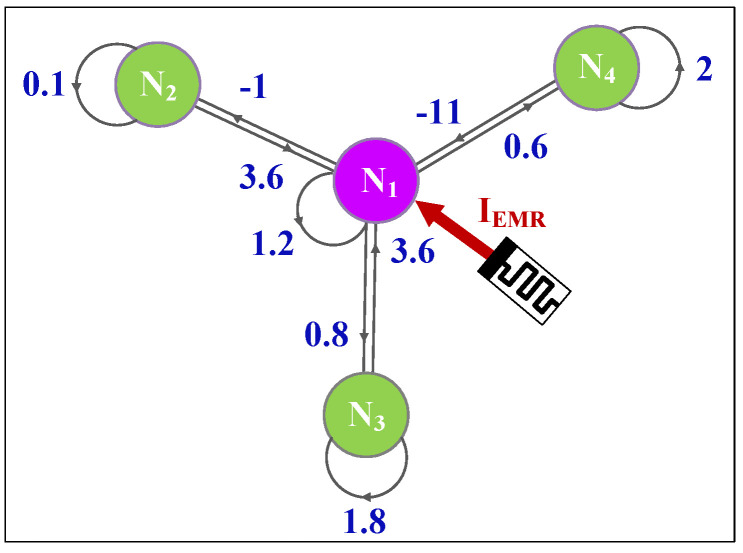
Concept map of the star memristive neural network.

A Hopfield neural network with a brain-like structure can be used to emulate chaotic behaviors of the biological nervous system. A Hopfield neural network with n neurons can be described by [1]

(3) $$C_{i}{\dot{x}}_{i}=-\frac{x_{i}}{R_{i}}+\sum_{j=1}^{n}w_{ij}\tanh(x_{j})+I_{i}\;(i,j\in N*)$$

where *C_i_*, *R_i_*, and *x_i_* are the membrane capacitance, membrane resistance, and membrane potential of the neuron *i*, respectively. *w_ij_* is the synaptic weight coefficient between neuron *j* and neuron *i*. In addition, tanh(.) denotes the neuron activation function, and *I_i_* is an external input current. Based on the original HNN (3), setting *C_i_* = 1, *R_i_* = 1, and *I_i_* = 0, a new HNN with four neurons is proposed as follows:

(4) $$\{\begin{array}{c} {\dot{x}}_{1}=-x_{1}+1.2\tanh(x_{1})+3.6\tanh(x_{2})+3.6\tanh(x_{3})-11\tanh(x_{4})\\ {\dot{x}}_{2}=-x_{2}-\tanh(x_{1})+0.1\tanh(x_{2})\\ {\dot{x}}_{3}=-x_{3}+0.8\tanh(x_{1})+1.8\tanh(x_{3})\\ {\dot{x}}_{4}=-x_{4}+0.6\tanh(x_{1})+2\tanh(x_{4}) \end{array}$$

where *x_i_* is the membrane potentials of neuron *i*. As shown in Figure 2, the four neurons are made up of a star neural network. A large number of research results show that the electromagnetic induction effect can be described by a flux-controlled memristor. According to the principle, the effect of external stimuli induced by electromagnetic radiation can be regarded as an additional forcing current IEMR. Consequently, when we consider that neuron 1 is stimulated by external electromagnetic radiation, the memristive star neural network can be modeled and described as follows:

(5) $$\{\begin{array}{c} {\dot{x}}_{1}=-x_{1}+1.2\tanh(x_{1})+3.6\tanh(x_{2})+3.6\tanh(x_{3})-11\tanh(x_{4})+\mu W(\varphi )x_{1}\\ {\dot{x}}_{2}=-x_{2}-\tanh(x_{1})+0.1\tanh(x_{2})\\ {\dot{x}}_{3}=-x_{3}+0.8\tanh(x_{1})+1.8\tanh(x_{3})\\ {\dot{x}}_{4}=-x_{4}+0.6\tanh(x_{1})+2\tanh(x_{4})\\ \dot{\varphi }=cx_{1}-dh \end{array}$$

where *φ* is the magnetic flux across the membrane of neuron 1, µ is the feedback intensity of external stimuli induced by electromagnetic radiation, and µ*w*(*φ*)*x*_1_ is the electromagnetic induction current.

### 2.3. Equilibrium Points and Their Stability

The equilibrium points of the star memristive neural network and their stabilities are analyzed in this subsection. Setting the left side of Equation (5) to 0, the equilibrium points are solved by

(6) $$\{\begin{array}{c} -x_{1}+1.2\tanh(x_{1})+3.6\tanh(x_{2})+3.6\tanh(x_{3})-11\tanh(x_{4})+\mu W(\varphi )x_{1}=0\\ -x_{2}-\tanh(x_{1})+0.1\tanh(x_{2})=0\\ -x_{3}+0.8\tanh(x_{1})+1.8\tanh(x_{3})=0\\ -x_{4}+0.6\tanh(x_{1})+2\tanh(x_{4})=0\\ cx_{1}-dh=0 \end{array}$$

Solving Equation (6), one can obtain

(7) $$\left\{x_{1}*,x_{2}*,x_{3}*,x_{4}*,\varphi *\right\}=\left\{0,0,0,0,h(\varphi *)=0\right\}$$

(8) $$\varphi *=\{\begin{array}{c} 0,\pm 2,\pm 4,\ldots ,(N)\\ \pm 1,\pm 3,\pm 5,\ldots ,(M) \end{array}$$

Obviously, there are infinite discrete equilibrium points. The Jacobian matrix of each equilibrium point can be calculated by

(9) $$\begin{array}{c} J=\left[\begin{array}{ccccc} -1+1.2s_{1}+\mu W\left(\varphi \right) & 3.6s_{2} & 3.6s_{3} & -11s_{4} & \mu x_{1}b\\ -s_{1} & -1+0.1s_{2} & 0 & 0 & 0\\ 0.8s_{1} & 0 & -1+1.8s_{3} & 0 & 0\\ 0.6s_{1} & 0 & 0 & -1+2s_{4} & 0\\ c & 0 & 0 & 0 & -dh^{\prime } \end{array}\right]\\ =\left[\begin{array}{ccccc} 0.2+\mu (a+b\varphi *) & 3.6 & 3.6 & -11 & 0\\ -1 & -0.9 & 0 & 0 & 0\\ 0.8 & 0 & 0.8 & 0 & 0\\ 0.6 & 0 & 0 & 1 & 0\\ c & 0 & 0 & 0 & -d \end{array}\right] \end{array}$$

where *s_i_* = sech^2^(*x_i_*). When setting *a* = 1, *b* = 0.01, *c* = 0.7, and *d* = 1.3, the corresponding eigenvalues and stabilities are given in Table 1. From Table 1, all the equilibrium points are of unstable saddle type. According to the Shilnikov theorem, the star memristive neural network may have a chaotic phenomenon.

## 3. Dynamic Analysis

In this section, chaotic dynamics of the proposed star memristive neural network are revealed by using nonlinear dynamics methods including bifurcation diagrams, Lyapunov exponents, phase plots, Poincaré maps, and basins of attraction. It should be noted that the numerical simulations are completed in MATLAB R2017a with the ODE45 algorithm. Meanwhile, the start time, the time step, and the time length are set as 500, 0.01, and 4000, respectively.

### 3.1. µ-Related Chaotic Dynamics

**Figure 3:**
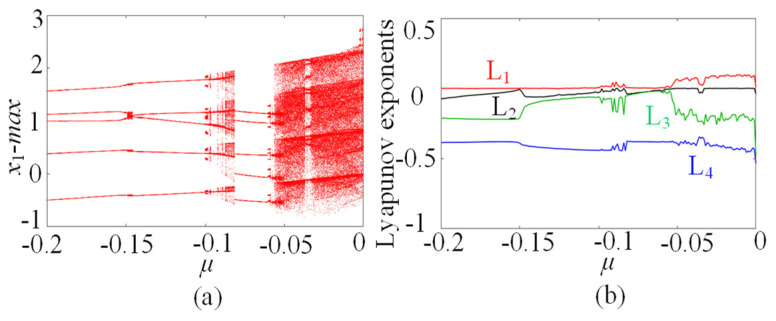
The µ-dependent dynamics with initial states (0.1, 0.1, 0.1, 0.1, 0.1). (**a**) Bifurcation diagram. (**b**) Lyapunov exponents.

**Figure 4:**
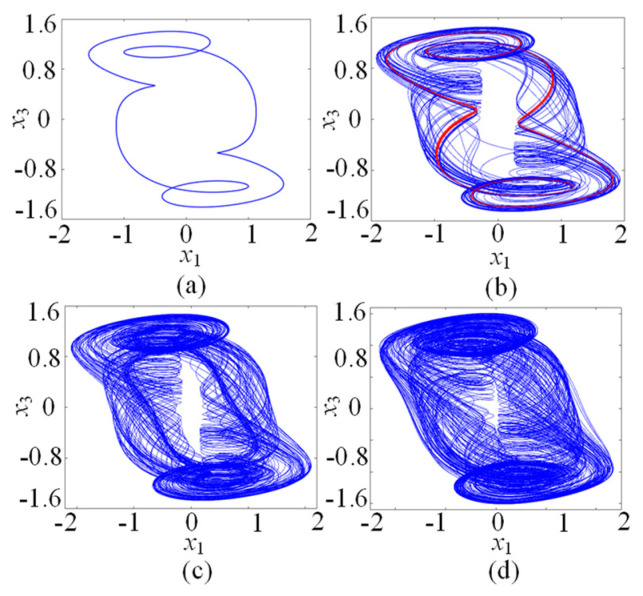
Dynamical behaviors related to µ. (**a**) Periodic attractor with µ = −0.2. (**b**) Transient chaotic attractor with µ = −0.08. (**c**) Chaotic attractor with µ = −0.05. (**d**) Chaotic attractor with µ = −0.01.

In this subsection, the chaotic dynamics related to the memristive coupling strength µ is analyzed. Setting the parameters *a* = 1, *b* = 0.01, *c* = 0.7, *d* = 1.3, *N* = 0, and initial conditions (*x*_10_, *x*_20_, *x*_30_, *x*_40_, *φ*_0_) = (0.1, 0.1, 0.1, 0.1, 0.1), the parameter µ increases from −0.2 to 0, and the µ-based bifurcation diagram is plotted in Figure 3a, where *x*_1_max is the maxima of the membrane potential *x*_1_. Moreover, the corresponding Lyapunov exponents are shown in Figure 3b. It can be seen from Figure 3 that the star memristive neural network can generate complex dynamical behaviors including period, transient chaos, and chaos. For example, with µ increasing from −0.2 to 0, the dynamical trajectory of the star memristive neural network starts from the period of entering into transient chaos at µ = −0.1, and then the transient chaotic behavior degrades into periodic behavior at u =−0.08. Interestingly, with the µ increasing to −0.055, the star memristive neural network enters chaos. It can be seen that the star memristive neural network exhibits a wide range of chaos until µ = 0. The phase portraits of the star memristive neural network with different values of u are given to illustrate its dynamical evolution with the parameter µ, as shown in Figure 4. It is obvious that the star memristive neural network successively produces periodic attractors, transient chaotic attractors, and chaotic attractors with the increase in *µ*.

### 3.2. N/M-Related Multi-Scroll Attractors

**Figure 5:**
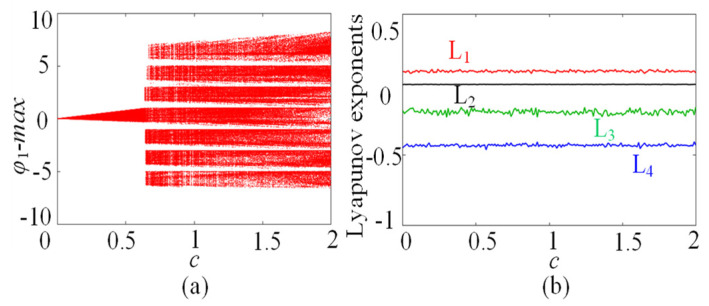
Dynamics of the SMNN with respect to the parameter *c*. (**a**) Bifurcation diagram; (**b**) Lyapunov exponents.

**Figure 6:**
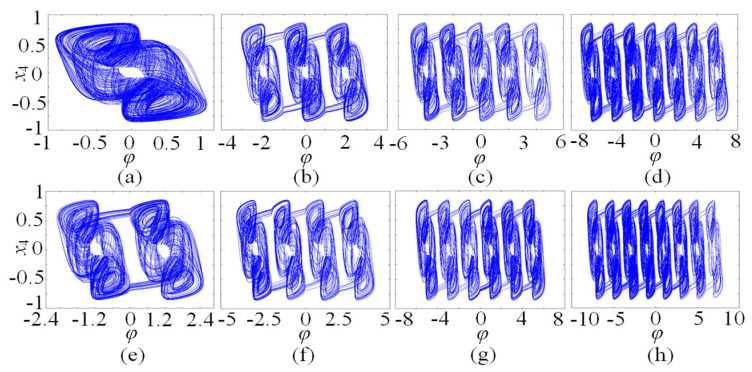
n-scroll attractors of the SMNN with different values of *N* and *M*. (**a**) 1-scroll attractor with *N* = 0; (**b**) 3-scroll attractor with *N* = 1; (**c**) 5-scroll attractor with *N* = 2; (**d**) 7-scroll attractor with *N* = 3; (**e**) 2-scroll attractor with *M* = 0; (**f**) 4-scroll attractor with *M* = 1; (**g**) 6-scroll attractor with *M* = 2; (**h**) 8-scroll attractor with *M* = 3.

In this subsection, the chaotic dynamics related to memristor control parameters N/M are analyzed. The numerical simulation shows that the SMNN can generate an arbitrary number of multi-scroll chaotic attractors related to N/M. When the parameters *a* = 1, *b* = 0.01, *d* = 1.3, *µ* = −0.01, *N* = 3, and initial states are kept unchanged, *c* is selected as the control parameter. A bifurcation diagram and the corresponding Lyapunov exponents related to parameter *c*∈(0, 2) are shown in Figure 5a and Figure 5b, respectively. Interestingly, it can be seen from the bifurcation diagram that the bifurcation diagram is made up of a dense patch of points with seven steps. This means that the SMNN not only generates chaotic attractors but also produces seven-scroll attractors. From the Lyapunov exponents, it shows one positive value, namely, it is chaotic behavior. Further research shows that the SMNN can generate an arbitrary number of multi-scroll attractors by selecting different control parameters N/M. To better understand the multi-scroll attractors, Figure 6 gives the phase portraits of multi-scroll attractors under different numbers of scrolls. Obviously, the number of scrolls generated by the star memristive neural network can be controlled by 2*N* + 1 and 2*M* + 2.

**Figure 7:**
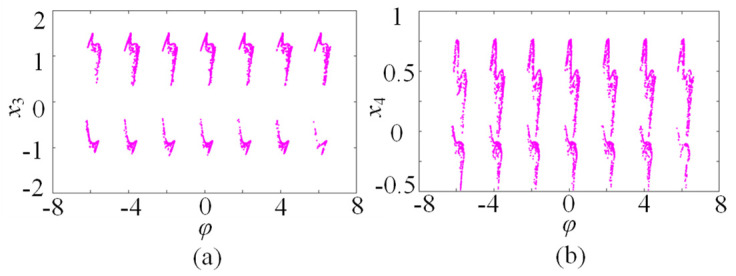
Poincaré maps of the 7-scroll attractor for x_1_ = 0. (**a**) on *φ*-*x*_3_ plane; (**b**) on *φ*-*x*_4_ plane.

In addition, the Poincaré maps on *φ*-*x*_3_ and *φ*-*x*_4_ phase planes for the 7-scroll attractor with *x*_1_ = 0 are depicted in Figure 7a and Figure 7b, respectively. Clearly, the Poincaré maps have approximately 7-scroll maps, implying that the star memristive neural network generates extremely complex multi-scroll attractors.

### 3.3. φ_0_-Related Initial Boosting Behavior

**Figure 8:**
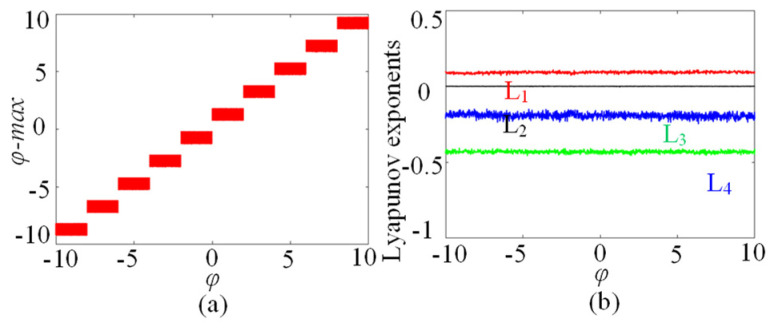
The φ_0_-dependent dynamics with the coupling strength *µ* = 0.5. (**a**) Bifurcation diagram. (**b**) Lyapunov exponents.

**Figure 9:**
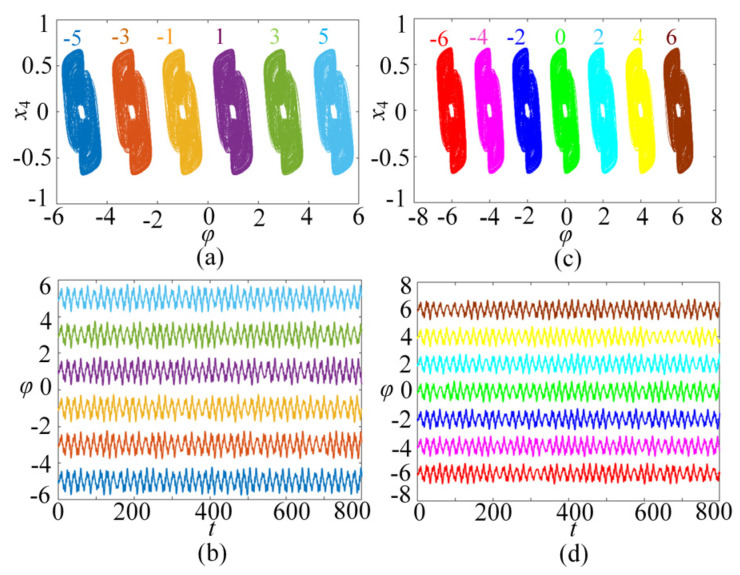
Coexisting multiple attractors in the SMNN with different φ_0_. (**a**) Six Coexisting attractors. (**b**) Corresponding time series. (**c**) Seven coexisting attractors. (**d**) Corresponding time series.

**Figure 10:**
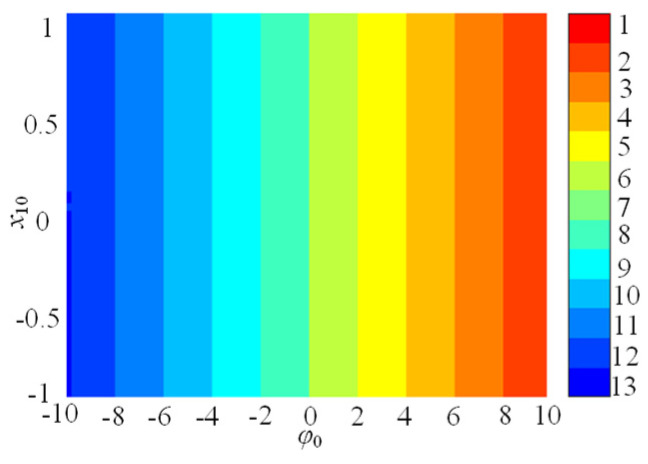
Basin of attraction for the SMNN.

In this subsection, the initial boosting chaotic phenomenon is analyzed. It is wonderful that the presented SMNN can generate initial boosting coexisting chaos. For instance, we plot the bifurcation diagram of the φ_0_ under *a* = 1, *b* = 0.01, *c* = 0.5, *d* = 1.3, *µ* = −0.01, *M* = 4, and *x*_10_ = *x*_20_ = *x*_30_ = *x*_40_ = 0.1, as shown in Figure 8a. As can be seen, the SMNN displays a complicated initial boosting behavior. Meanwhile, the corresponding constant Lyapunov exponents in the whole range of the *φ*_0_ variation are given in Figure 8b. Obviously, the SMNN has an infinite wide chaotic range along the *φ*_0_-axis. That is to say, the SMNN enjoys complex dynamics of initial boosting coexisting chaos, which means that it has excellent robustness. Moreover, to further verify initial boosting dynamics, the local attraction basin in the *φ*_0_-*x*_10_ plane is given in Figure 9. From Figure 10, the local attraction basin has complex manifold structures and basin boundaries, and the specified initial value regions are composed of different colored zones marked by 1–13, among which the colored zones marked by 1–13 correspond to the attractors with different positions. Therefore, the star memristive neural network has complex initial boosting behavior.

## 4. Circuit Validation

With the rapid development of artificial intelligence, the physical realization of neural network models is very important for developing neuromorphic hardware systems [42,43]. It is a reliable way to realize neural network models through analog circuits. This is because the analog neural network circuit can not only achieve real-time calculation but also reproduce the behavior of the real nervous system. In this section, the proposed star memristive neural network model is realized by using basic electronic circuit elements such as resistors, capacitors, operational amplifiers, and analog multipliers. We first design the circuit of the star memristive neural network model and verify its complex behavior in Multisim.

### 4.1. Design of the SMNN Circuit

**Figure 11:**
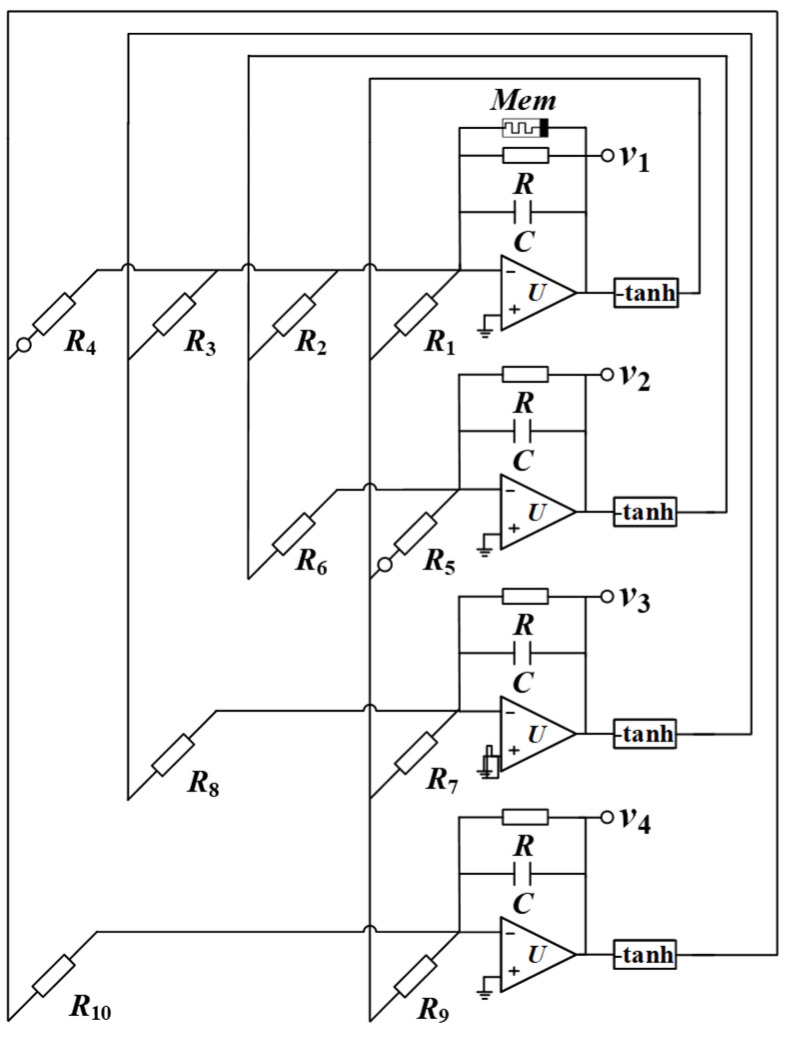
Circuit implementation.

Before implementing the neural network circuit, we first introduce a hyperbolic tangent excitation function circuit [23] and a memristor circuit [15], respectively. Based on the two circuit units, a memristive Hopfield neural network circuit can be designed. According to the star memristive neural network model (5), the circuit structure is designed in Figure 11. Four membrane potentials *x*_1_, *x*_2_, *x*_3_, and *x*_4_ are emulated by four out voltages *v*_1_, *v*_2_, *v*_3_, and *v*_4_, respectively. All synaptic weight coefficients are simulated by the resistors *R*_1_-*R*_10_. Based on the circuit in Figure 11, the circuit state equations can be described by

(10) $$\{\begin{array}{c} RC\frac{dv_{1}}{dt}=-v_{1}+\frac{R}{R_{1}}\tanh(v_{1})+\frac{R}{R_{2}}\tanh(v_{2})+\frac{R}{R_{3}}\tanh(v_{3})-\frac{R}{R_{4}}\tanh(v_{4})-\left(\frac{R}{R_{a}}+\frac{R}{R_{b}}gv_{\varphi }\right)v_{1}\\ RC\frac{dv_{2}}{dt}=-v_{2}-\frac{R}{R_{5}}\tanh(v_{1})+\frac{R}{R_{6}}\tanh(v_{2})\\ RC\frac{dv_{3}}{dt}=-v_{3}+\frac{R}{R_{7}}\tanh(v_{1})+\frac{R}{R_{8}}\tanh(v_{3})\\ \begin{array}{c} RC\frac{dv_{4}}{dt}=-v_{4}+\frac{R}{R_{9}}\tanh(v_{1})+\frac{R}{R_{10}}\tanh(v_{4})\\ RC\frac{dv_{\varphi }}{dt}=\frac{R}{R_{c}}v_{1}-\frac{R}{R_{d}}h\left(v_{\varphi }\right) \end{array} \end{array}$$

Assuming that *C*_1_ = *C*_2_ = *C*_3_ = *C*_4_ = *C* = 1 nF, *RC* = 10 us, and resistance *R* = 10 kΩ, then *C* can be chosen as 1 nF. Considering the fixed synaptic weight coefficients, part resistors can be calculated as *R*_1_ = 8.3 kΩ, *R*_2_ = 2.7 kΩ, *R*_3_ = 2.7 kΩ, *R*_4_ = 0.9 kΩ, *R*_5_ = 10 kΩ, *R*_6_ = 100 kΩ, *R*_7_ = 12.5 kΩ, *R*_8_ = 5.5 kΩ, *R*_9_ = 16 kΩ, and *R*_10_ = 5 kΩ. In addition, *Ra* = *R*/*aµ*, *Rb* = *Rg/bµ*, *Rc* = *R/c*, and *Rd* = *R/d* are adjustable resistors. In the above formula, *g* represents the factor of the multiplier M. In this circuit, g is equal to 0.1.

### 4.2. Measurement of the SMNN Circuit

**Figure 12:**
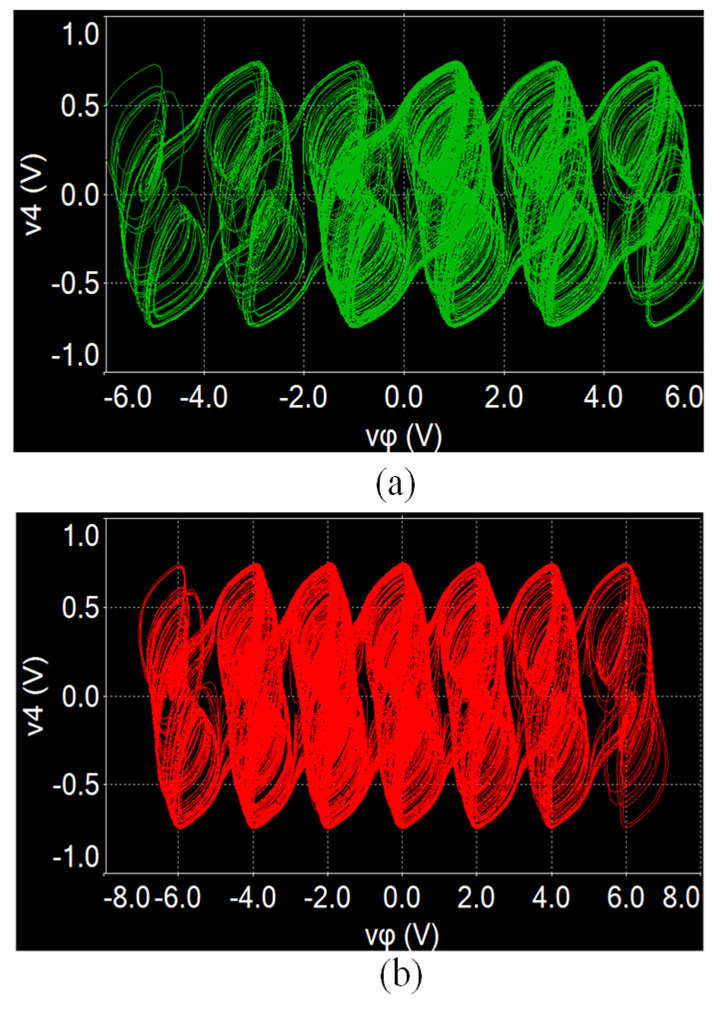
Experimentally captured multi-scroll attractors from the SMNN circuit with *Rc* = 13.9 kΩ. (**a**) Six-scroll attractor. (**b**) Seven-scroll attractor.

**Figure 13:**
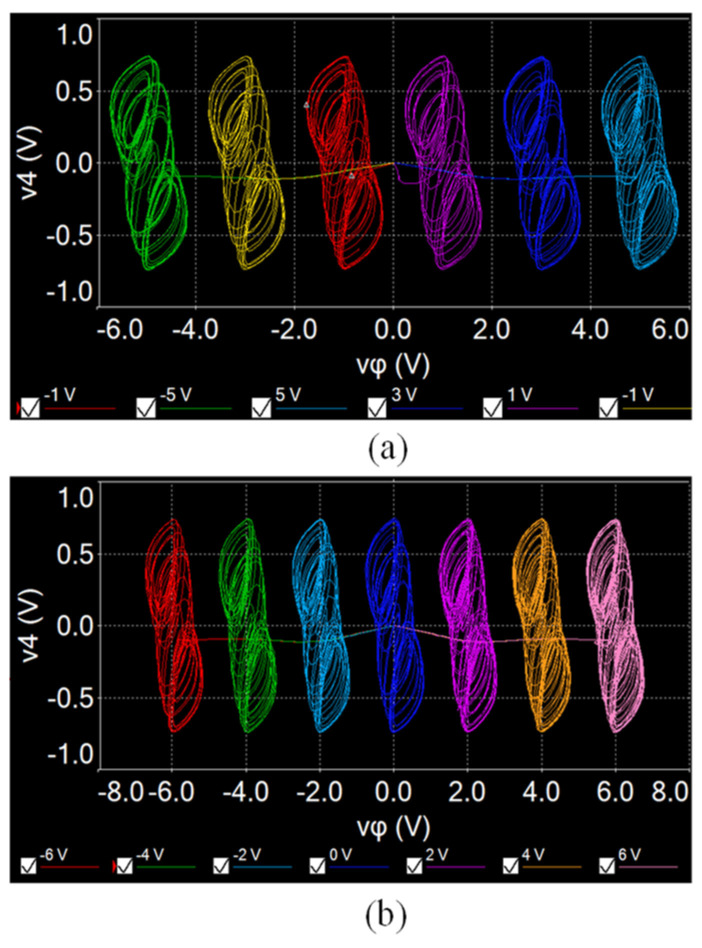
Experimentally captured coexisting attractors from the SMNN circuit with *Rc* = 20 kΩ. (**a**) Six coexisting attractors. (**b**) Seven coexisting attractors.

The designed star memristive neural network circuit is verified by using the MULTISIM platform. When *a* = 1, *b* = 0.01, *µ* = 0.01, *c* = 0.7, *d* = 1.3, and initial values (0.1, 0.1, 0.1, 0.1, 0.1), and setting *Ra* = 1 MΩ, *Rb* = 10 MΩ, *Rc* = 13.9 kΩ, *Rd* = 8 kΩ, and initial capacitor voltages to 0.1 V, multi-scroll attractors can be generated by selecting different switches. For example, closing W_1_, W_2_, and W_3_ and setting *e*_1_ = 2 V and *e*_2_ = 4 V, a six-scroll chaotic attractor can be generated from the designed circuit, as shown in Figure 12a. But when changing *Rc* = 20 kΩ (*c* = 0.5), the initial boosting coexisting attractors can be realized by adjusting different initial capacitor voltages *v_φ_*_0_. As shown in Figure 12b, six coexisting chaotic attractors are obtained from the neural network circuit. Similarly, keeping *Rc* = 13.9 kΩ; closing W_2_, W_3_, and W_4_; and setting *e*_1_ = 1 V, *e*_2_ = 3 V, and *e*_3_ = 5 V, a seven-scroll chaotic attractor can be obtained, as shown in Figure 13a. Keeping *Rc* = 20 kΩ, seven coexisting chaotic attractors can be obtained as shown in Figure 13b. It should be noted that the circuit simulation results are slightly different from the numerical results because of the computing errors between two different tools.

## 5. Application in a Color Cryptosystem

Chaos can be used for information encryption due to its high randomness and sensitivity [44,45]. Chaotic neural networks with complex dynamic behavior have more promising applications for information encryption [46,47,48]. In this section, a new color image encryption scheme is designed based on the proposed star memristive neural network with multi-scroll attractors.

The encryption and decryption process is described in the following steps.

Step 1: Setting *a* = 1, *b* = 0.01, *c* = 0.7, *d* = 1.3, *µ* = 0.01, *N* = 3, and initial states (0.1, 0.1, 0.1, 0.1, 0.1), the proposed SMNN is iterated N0 + mn times, and the last mn iterations are regarded as valid data. Each iteration can generate five chaotic seven-scroll sequences *x*_1_(*i*), *x*_2_(*i*), *x*_3_(*i*), *x*_4_(*i*), and *φ*(*i*), where *i* = [1, mn].

Step 2: To obtain a pseudo-random sequence, the generated sequences are preprocessed as

(11) $$s(i)=\mathrm{mod}(\mathrm{floor}((\left|x_{1}(i)\right|+\left|x_{2}(i)\right|+\left|x_{3}(i)\right|+\left|x_{4}(i)\right|+\left|\varphi (i)\right|)*10^{7}),256)$$

where mod(*x*) is the modulo operation and floor(*x*) is the flooring operation.

Step 3: A chaotic sequence is generated, which can be described as

(12) K(i)=[s(1),s(2),s(3),…,s(mn)]

Step 4: *K*(*i*) is used to encrypt the original image using the XOR operation, as follows:

(13) C(i)=P(i)⊕K(i)

To demonstrate the efficiency of the designed image encryption cryptosystem, three color images “Lena”, “virus”, and “chameleon” with a size of 512 × 512 are chosen as the encryption object. The experimental results and the security performance analyses including the histogram, correlation coefficient, information entropy, key sensitivity, data loss, and noise attacks are given in the following.

**Figure 14:**
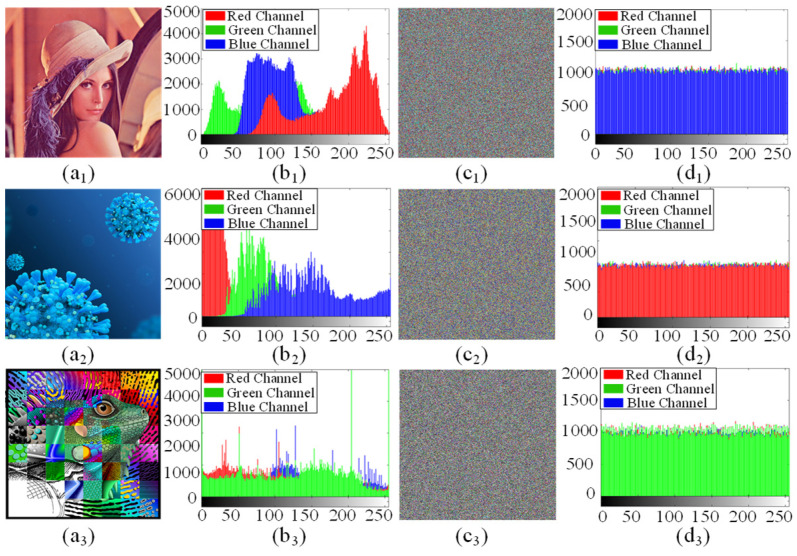
Encryption results of the proposed encryption scheme: (**a1**–**a3**) original images; (**b1**–**b3**) histograms of the original images; (**c1**–**c3**) encrypted images; (**d1**–**d3**) histograms of the encrypted images.

(1) Histogram analysis:

Histograms are used to evaluate the distribution of pixel intensity values in an image. In theory, a good image encryption system should produce a uniform histogram. Figure 14 gives the original images, the encrypted images, and their respective histograms. Obviously, the encrypted images in Figure 14(c1) look cluttered and completely lose their original information. The histograms of the encrypted images in Figure 14(d1–d3) are almost uniform, which means that it is difficult to obtain any useful statistical information from the encrypted image. Therefore, the proposed image encryption scheme is enough to resist statistical attacks.

(2) Correlation analysis: The relationship between adjacent pixels in an image can be described using correlation. In general, a stronger correlation indicates a more regular image and a weaker correlation indicates a more chaotic image. The correlation coefficient is computed by [45]

(14) $$\rho_{xy}=\frac{\sum_{i=1}^{N}\left(x_{i}-E(x))(y_{i}-E(y)\right)}{\sqrt{\sum_{i=1}^{N}{\left(x_{i}-E(x)\right)}^{2}}\sqrt{\sum_{i=1}^{N}{\left(y_{i}-E(y)\right)}^{2}}}$$

where *x* and *y* are the intensity values of two adjacent pixels. Here, 10,000 pairs of pixels were randomly chosen in horizontal, vertical, and diagonal directions from the original image and corresponding encrypted image to evaluate the correlation coefficient. As shown in Table 2, after the original images were encrypted, their relevance was greatly reduced. Therefore, the designed cryptosystem has a strong ability to resist statistical attacks.

(3) Entropy analysis: Information entropy can be used to evaluate the randomness of image information. The information entropy is calculated as [45]

(15) $$H(P)=\sum_{i=0}^{2^{N}-1}P(x_{i})\log_{2}\frac{1}{P(x_{i})}$$

where *P*(*x_i_*) denotes the probability of *x_i_* and 2*N* represents the number of the information source. The maximum theoretical information entropy is 8. Table 3 gives the calculation results of information entropy under different channels. From the results in Table 3, it can be seen that compared with other similar schemes, the information entropy of this scheme is closer to the theoretical value.

**Figure 15:**
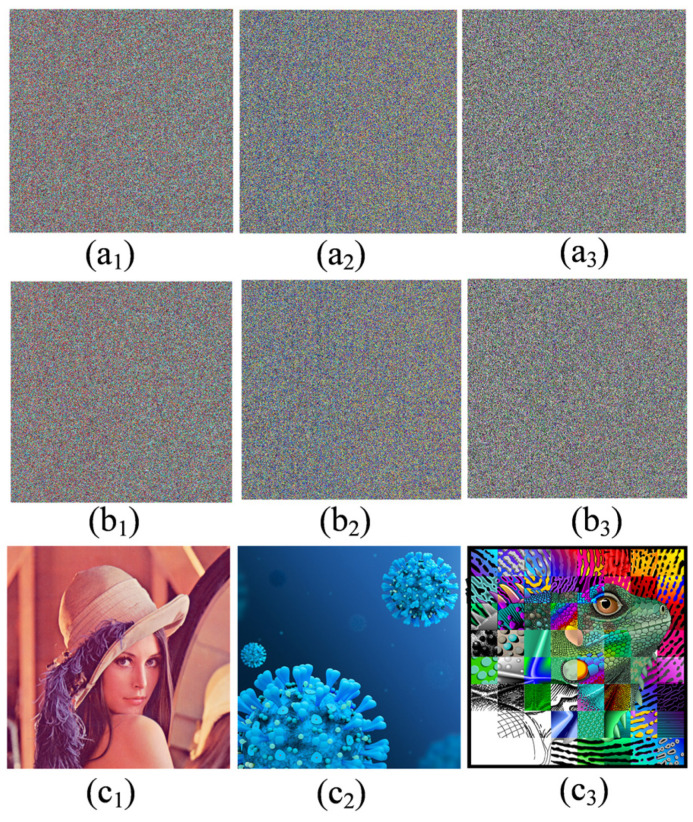
Sensitivity test results with the secret key (*x*_10_, *x*_20_). (**a1**–**a3**) Inaccurate decrypted image with an inaccurate secret key *x*_10_ = 0.1 + 10^−16^; (**b1**–**b3**) inaccurate decrypted image with an inaccurate secret key *x*_20_ = 0.1 + 10^−16^; (**c1**–**c3**) accurate decrypted image with the secret keys (0.1, 0.1).

(4) Sensitivity analysis: Key sensitivity is an important indicator for measuring the security of encryption algorithms. A good image encryption scheme should be key-sensitive. The initial values are used as secret keys in this encryption algorithm. The decrypted images are shown in Figure 15(a1–b3) with a slight change of the secret key. Despite the fact that the secret key has been changed a little (10–16), the decrypted images are completely different from the original image. Figure 15(c1–c3) shows the decrypted image with the correct secret key. As shown in Table 4, compared with other similar image encryption schemes, the image encryption scheme proposed in this paper has a higher sensitivity to the key.

(5) Data loss and noise attacks

**Figure 16:**
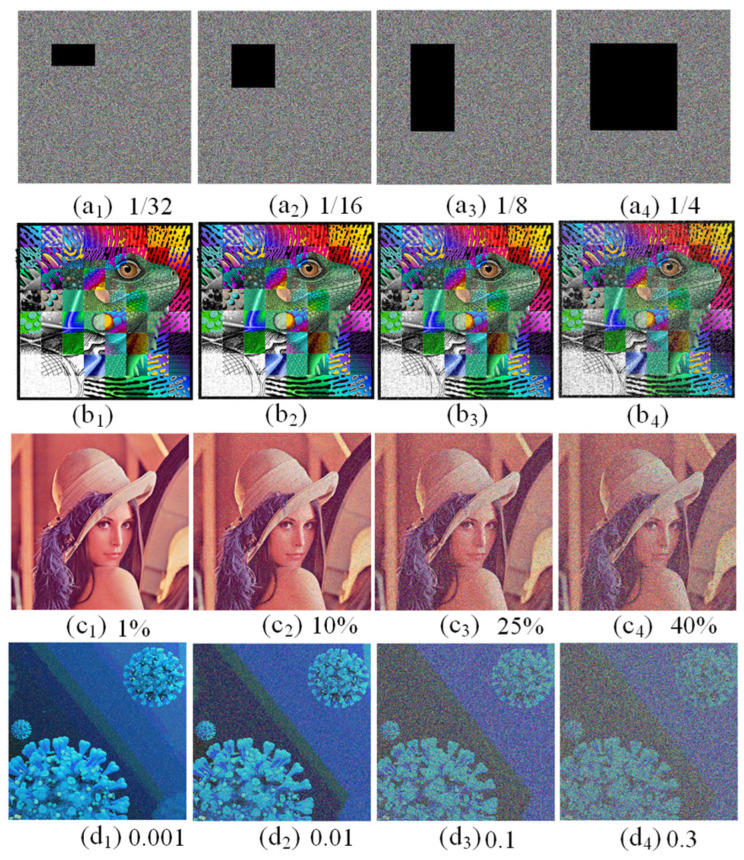
The test results of the proposed scheme for data loss and noise attacks. (**a1**–**a4**) The encrypted image with data loss. (**b1**–**b4**) Corresponding decrypted images. (**c1**–**c4**) The decrypted images under salt and pepper noise. (**d1**–**d4**) The decrypted images under Gaussian noise.

Data loss and noise attacks can seriously affect the decryption effect of encrypted images. To evaluate the ability of data loss and noise attacks, we cut off some parts of the encrypted image and then decrypt it. As shown in Figure 16(a1–b4), data loss attacks for the different lost areas are successfully decrypted for the original image to be recovered. In order to test the resistance of the algorithm to noise attack, salt and pepper noise with different concentrations is added to the encrypted images. It can be seen from Figure 16(c1–c4) that some pixel values in the decrypted images have been changed; however, the approximate information of the original image can still be recovered successfully. Furthermore, when different concentrations of Gaussian noise are added to the encrypted images, these images are still able to be decrypted, as shown in Figure 16(d1–d4). Consequently, we can conclude that the proposed color image encryption scheme is able to withstand data loss and noise attacks and has high security.

## 6. Conclusions

In this article, the chaotic dynamics of a star memristive neural network are studied. First, based on a Hopfield neural network with four neurons and a flux-controlled memristor, a star memristive neural network model is constructed. Then, its chaotic behaviors are revealed by using various numerical methods. Analysis results show that the star memristive neural network can generate abundant chaotic dynamics including chaos, multi-scroll attractors, and initial boosting coexisting behavior. Especially, the number of scrolls for the multi-scroll attractors can be changed by adjusting the memristor’s control parameters. In addition, the position and number of coexisting attractors can be changed by switching the memristor’s initial value. To further verify these results, an analog neural network circuit is designed and implemented. All numerical results are experimentally verified by MULTISIM circuit simulation. Finally, a color image encryption scheme based on the proposed star memristive neural network is designed. Simulation results such as histograms, correlation, information entropy, key sensitivity, and data loss and noise attacks demonstrate that the designed image encryption scheme has good security.

With the rapid development of memristors, memristor-based neural networks have been widely used in various fields including memristive neurodynamics [51,52], memristive neuromorphic computation [53,54], and so on [55]. In future work, we will devote ourselves to studying the chaotic dynamics of the memristive neural networks with different topology structures. We will also explore the practical applications of the memristive neural network developed here.

## Figures and Tables

**Table 1:** Equilibrium points and their stabilities.

Equilibrium Points	Eigenvalues	Stability
(0,0,0,0,0)	(−1.3,0.1789 ± 2.5068i,0.162,0.5698)	Unstable saddle-focus
(0,0,0,0,1)	(−1.3,0.1789 ± 2.5068i,0.162,0.5698)	Unstable saddle-focus
(0,0,0,0,−1)	(−1.3,0.1791 ± 2.5068i,0.1619,0.5699)	Unstable saddle-focus
(0,0,0,0,2)	(−1.3,0.1790 ± 2.5068i,0.1621,0.5698)	Unstable saddle-focus
(0,0,0,0,−2)	(−1.3,0.1791 ± 2.5068i,0.1619,0.5699)	Unstable saddle-focus

**Table 2:** Correlation coefficients for different images.

Images	Type	Horizontal	Vertical	Diagonal
Lena	Original	0.9874	0.9775	0.9720
	Encrypted	0.0017	0.0138	0.01803
Virus	Original	0.9837	0.9846	0.9772
	Encrypted	0.0028	0.0085	0.0103
Chameleon	Original	0.8777	0.8905	0.8116
	Encrypted	0.0033	0.0024	0.0099

**Table 3:** Information entropy in different signal channels and different encryption schemes.

Refs	RGB	Red	Green	Blue
[46]	7.9993	7.9993	7.9994	7.9993
[47]	7.9994	7.9994	7.9993	7.9993
[49]	7.9991	7.9972	7.9967	7.9985
Lena	7.9998	7.9994	7.9993	7.9994

**Table 4:** Key sensitivity in different encryption schemes.

Refs	[31]	[48]	[50]	[49]	This Work
Key sensitivity	10^−15^	10^−14^	10^−14^	10^−14^	10^−16^

## Data Availability

Please contact the corresponding author.
